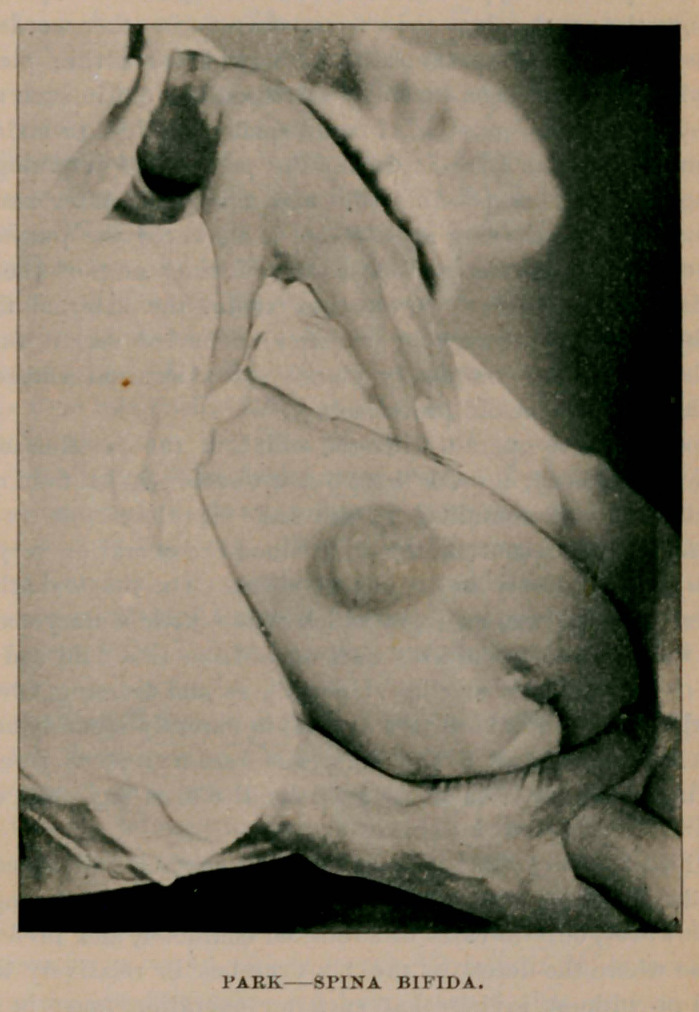# A Case of Spina Bifida Treated by Operation, Including Closure of the Spinal Canal by a Celluloid Plate

**Published:** 1895-08

**Authors:** Roswell Park

**Affiliations:** Professor of surgery, Medical Department, University of Buffalo. 510 Delaware Avenue


### A CASE OF SPINA BIFIDA TREATED BY OPERATION, INCLUDING CLOSURE OF THE SPINAL CANAL BY A CELLULOID PLATE.

By ROSWELL PARK, A. M., M. D.,
Professor of surgery, Medical Department, University of Buffalo.

KATE STIGLER, *æt.* 5 months, was brought to my clinic at Buffalo General Hospital by Dr. Hitzel, of Buffalo, in March, 1895. As the accompanying illustration will show, there was a thin-walled, elastic, fluctuating tumor located centrally over the lower part of the spinal column. So thin were its walls that on holding the child up to the light the tumor proved translucent, as does a hydrocele when similarly treated. It was verynearly the size of a split hen's egg. Evidently a spina bifida, the tumor consisted apparently of a meningeal protrusion covered simply by integument, although around its base there was some fatty tissue, irregularly distributed. Even in front of a powerful light

I could not definitely make out whether any nerve trunks were adherent to its external aspect or not. Patient's general condition excellent, there being neither ulceration nor abrasion of the skin in the neighborhood. The tumor was growing relatively considerably faster than the child, and, since there was no present infection of its sac nor any imminent danger of it, I proposed operation, to which the parents consented.

PARK—SPINA BIFIDA.

This was made March 18, 1895, in my clinic, the child being anesthetised with ether. The operation consisted in complete extirpation of the external portion of the sac, its central and most protruding area being excised by an elliptical incision. The neck of the sac was found relatively small, and the defect in the bone involved apparently three of the infant's vertebral arches. Projecting into the general cystic cavity was a polypoid protrusion, composed apparently of spinal pia and arachnoid. This arrangement, then, was of one sac projecting into another, in which it floated. Inasmuch as this inner sac contained no visible nerves, nothing but membrane and fluid, its neck was ligated at its exit from the spinal canal, the protruding part excised, and the ligated stump tucked back into the canal. Into the osseous defect—in other words, into the opening into the spinal canal—I now fitted a thin piece of celluloid which had been carefully sterilised and cut in such a way that, by a little manipulation, I could spring its edges in under the bony margin, which I freshened for the purpose of retaining and holding as firmly as possible this new material. After carefully inserting it and satisfying myself that it answered the purpose of a posterior wall perfectly, I closed over it the stump of the main sac with catgut sutures. Over this, again, the external tissues were held firmly together by a series of buried catgut sutures, until the wound was completely closed and a straight suture line took the place of the old protrusion.

I took pains to operate with the child in the position of the down-hanging head, in order that no more cerebro-spinal fluid might be lost than was contained in the sac. As the result, in some measure, of this precaution, the child stood the operation very well and displayed scarcely any signs of shock. On the seventh day the first dressing was made, at which time a little watery accumulation was released. Cultures were made from this fluid and were found to be absolutely sterile. Upon the second dressing, five days later, the parts were absolutely dry and apparently securely healed. There has never been a bad sign, and recent reports from the child indicate that it is in as good condition as any child of its age.

This case report will illustrate one of the recent and successful methods of dealing with a serious congenital lesion. It is applicable, however, only to cases of a selected character, and preferably to those where the defect in the bony arches is relatively small. It will go without saying that such an operation must be done under rigid aseptic precautions, to which end it is vastly preferable to get hold of such a case before there has been any ulceration of the skin, or anything like the formation of pressure sores. The operation of excision of the tumor is by no means new, but has been done many times. Mayo Robson has succeeded in a number of instances, as have others, in transplanting into the osseous defect periosteum with its entangled osteoblasts, from the bones of a rabbit or other animal. Osteoplastic operations have also been suggested and devised by other operators, all of which entail more handling and more uncertainty of result. A *résumé* of the various methods of attacking these cases, with allusion to a relatively large number of cases with results, was published by Marcy in the *Annals of Surgery* for March, 1895. Until very recently, however, it has not been thought feasible to insert into the spine plates of protective material,—metal, celluloid, etc.,—as has often been done within the cranium.

For the purpose of closing cranial defects, both heteroplastic and autoplastic methods have been described by many surgeons. In 1820, Walther replaced a resected portion of the skull with partial healing. In 1868, Wolff attained pretty uniform success in reimplanting bone in animals. Macewen, Weir and others then recommended the sewing of bone fragments over the dura. This has been practically discontinued. Then transplantation experiments were made with different animals, and in 1889 Seydel transplanted bone from the tibia of a patient to the skull, replacing that which had been lost as the result of a compound injury. Poncet resorted to bone removed from the skull of a newborn infant dead of asphyxia. Decalcified bone was suggested by Senn and by Kummel. Hinterstoisser reported most encouraging success with celluloid. Gerstein has employed rabbit bone and ivory, and aluminum and other materials have been recommended by yet others.

Following Beach, of Boston, I have a number of times inserted gold-foil between the pia and the dura to prevent formation of adhesions at this point. A thicker specimen of the same metal might easily be used for the purposes of reinforcing or affording strength. Strange stories have been circulated among the laity as to the use in time past, even centuries ago, of metal and glass plates for the same purpose, but these stories are almost invariably creations of a vivid imagination. Certain it is, however, that within the past few years we have learned that various foreign materials, sterilised and made inert, can be introduced within the human body for certain purposes among which are those herein indicated. Probably the most generally serviceable will be celluloid, of the use of which I herewith record an example. There is every reason, in the case I have described, to expect the perfect encapsulation of this plate and its incorporation into the tissues to an extent permitting of the perfect performance of the duty which I thus imposed upon it.

510 Delaware Avenue.

## Figures and Tables

**Figure f1:**